# Omega-3 Fatty Acid Fortification of Plant-Based Beverages to Enhance Their Nutritional Profile

**DOI:** 10.3390/foods14091602

**Published:** 2025-05-01

**Authors:** Ashish Pandey, Fozia Kamran, Manisha Choudhury, Li Li, Mohammad Shafiur Rahman, Malik Altaf Hussain

**Affiliations:** 1 School of Science, Western Sydney University, Hawkesbury Campus, Richmond, NSW 2753, Australiamanisha.choudhury@aau.ac.in (M.C.);; 2 Department of Food Science and Nutrition, College of Community Science, Assam Agricultural University, Jorhat 785013, India; 3 Department of Food Science and Nutrition, College of Agricultural and Marine Sciences, Sultan Qaboos University, P.O. Box q34-123, Al-Khod 123, Oman; shafiur@squ.edu.om

**Keywords:** plant-based beverage, nutritional value, omega-3 fatty acid, fortification, health benefits

## Abstract

The growing popularity of a diverse range of plant-based beverages is entrenched in promoting health functionality and addressing ethical and environmental concerns. These beverages offer similar physico-chemical attributes to animal milk and are prepared using plant-based ingredients, such as soy, oats, almonds, rice, chickpeas, sesame seeds, and coconut. These beverages have many nutritional benefits but are deficient in certain nutrients such as essential amino acids, minerals, vitamin B12, vitamin D, and omega-3 fatty acids. Fortifying these beverages with deficient nutrients could effectively provide comprehensive and nutritionally balanced product options. This approach could be useful in improving the nutritional profile of plant-based beverages to meet the expectations of health-conscious consumers. However, fortifying these products poses challenges related to taste, stability, and ingredient sourcing. Omega-3 fatty acids are crucial for human health and provide numerous health benefits, such as improved heart and vascular health, reduced inflammation, and the prevention of various health conditions. As plant-based diets gain popularity, the demand for nutritionally balanced products is growing, making omega-3 fortification a strategic approach for businesses to tap into an expanding market of health-conscious consumers. However, it is important to consider individual needs about health and ensure regulatory oversight to ensure the safety and effectiveness of fortified plant-based products. This article provides an overview of emerging plant-based beverages, their comparative nutritional profiles, the need to improve the nutritional value using omega-3 fatty acids as an example, and challenges in omega-3 fatty acid fortification.

## 1. Introduction

Plant-based beverages are prepared mainly from plant-based ingredients and have gained popularity in recent years due to various health, ethical, and environmental reasons, as well as religious beliefs. The numerous nutritional benefits of plant-based beverages have made them a good option for vegetarians and people with lactose intolerance or animal milk allergy [1,2]. Plant-based diets have gained traction primarily because of their assumed role in reducing the carbon footprints of the diets, prioritizing human well-being, and addressing concerns about animal welfare. While vegetarians and vegans constitute a smaller percentage of the population compared to omnivores, their ranks have seen a notable increase in recent times [3]. Some common types of plant-based beverages are produced using almonds, soy, oats, coconut, and rice. The research focus continues to explore the use of a myriad of legumes, seeds, nuts, and cereals in the preparation of plant-based beverages to attain their nutritional benefits. These products are not always an alternative to dairy milk, but innovative and nutritious drinks prepared with plant-based ingredients that have the potential to replace many alcoholic and non-alcoholic beverages available in the market or prepared domestically [1,2]. The sensory aspects, such as the color and texture of plant-based beverages, are considered close to animal-based milk, but nutritional aspects are far different. Plant-based beverages are low in calcium, protein (specific essential amino acids), and fat content, with some exceptions, but contain many micronutrients and bioactive compounds that help maintain human health [4]. The use of specific plant-based beverages should be considered carefully by consumers with specific conditions; for example, rice beverages have a high glycemic index, and soy beverages are known for their immunogenicity. Compared with cow’s milk, plant-based beverages seem to contain higher fiber content, some variably richer in a few non-calcium minerals, vitamin B, fat-soluble vitamins, and antioxidants that maintain the body’s physiological functions as well as protect against cancer, Alzheimer’s disease, diabetes, and cardiovascular illnesses [5,6]. The nutritional differences between dairy milk and plant-based beverages imply that the promotion and transition to plant-based beverages require a further improved nutritional profile in such products, particularly for those replacing them with dairy products [6,7,8]. Additionally, consumers’ education and dietary guidelines may include more information about how to strategically incorporate plant-based alternatives in diets to minimize the risk of deficiencies in key micronutrients, similar to those consistently found in populations following vegan or vegetarian diets with limited food diversity [9,10,11].

Innovation and development of fortified plant-based beverages is one convenient strategy to provide a complete nutritious drink for people with or at risk of deficiencies of vitamin D, calcium, and some essential minerals, and to help lower the risk of chronic diseases. For example, plant-based beverages fortified with omega-3 fatty acids could provide numerous benefits for maintaining brain, heart, and eye health, reducing aging problems, controlling diabetes, improving bone and nail health, and protecting against cancer [1,12]. Omega-3 fatty acids are mostly obtained from fish; however, seaweed, microalgae, flaxseeds, walnuts, and eggs also contain omega-3 fatty acids, but in lower quantities. It is widely recognized that in humans, the slow conversion of plant-sourced omega-3 fatty acid, alpha-linolenic acid (ALA, C18:3ω3), to long-chain omega-3 fatty acids, such as eicosapentaenoic acid (EPA; C20:5ω3) and docosahexaenoic acid (DHA; C22:6ω3), is insufficient to address requirements for health in general [12]. Therefore, it is essential to consume EPA and DHA from dietary sources, primarily fish oil, in the current food supply. The daily minimum requirement of long-chain omega-3 fatty acids is 0.5 g to maintain good health, but the amount recommended differs between countries and for achieving different health benefits [12,13]. This article uses the example of omega-3 fatty acid fortification to improve the nutritional profile of plant-based beverages and make them attractive to consumers with specific needs or preferences.

## 2. Nutrition Profile of Plant-Based Beverages

Plant-based beverages have diverse macro- and micronutrient profiles, as well as bioactive substances and antinutritional elements [14]. For example, soy-based beverages contain isoflavones and phytosterols, while almond-based beverages contain α-tocopherol and arabinose, and oat-based beverages have β-glucan. Regarding antinutritional factors, oxalates and phytates are present in the sesame-based beverage, while the oat-based beverage contains phytates as well [14]. Table 1 provides reported information on the variation (range) of macronutrients (carbohydrates, protein, fat, and dietary fiber) in selected plant-based beverages, such as oat milk, soy milk, almond milk, and others, available in 13 different countries (Table 1). To explore the different quantities of essential macronutrients in beverages made from the same variety of plants, the range of each nutrient is extracted from previous studies [4,15].

The cow’s milk has been considered standard when comparing the nutrients of all plant-based beverages. It has been found that most of the plant-based beverages contain less nutrient content compared with the same serving size as cow’s milk. Macronutrient information collected in Table 1 shows that cow’s milk is relatively high in energy, protein, and fat, making it a nutrient-dense option, but lacks dietary fiber. Plant-based beverages provide higher dietary fiber content than cow’s milk. Soy milk offers higher energy, higher carbohydrates, and good protein content comparable to many other plant-based beverages. Coconut milk contains the highest fat content, which provides high energy to the body. Rice milk is rich in carbohydrates and provides very little protein and fat content. Oat milk provides the highest dietary fiber and average protein content among all samples observed, making it one of the healthier options as compared to other plant-based beverages. Cashew and almond milk contain higher fat content and comparable carbohydrates compared to cow’s milk. Hemp milk is low in most of the macronutrients compared to cow’s milk and other plant-based beverages.

Each plant-based beverage offers a unique nutritional profile. Table 2 shows the range of micronutrient contents of different plant-based beverages reported in different studies.

The average (range) of essential minerals and vitamins of plant-based beverages is previously studied by Walther et al. [6]. Out of 27 samples of plant-based beverages available in Switzerland, some were fortified with calcium, vitamin D, and B12; otherwise, plant-based beverages lack those micronutrients. Calcium is essential for bone and dental health, the function of muscles, nerves, and blood vessels, and protection from osteoporosis. Most of the plant-based beverages are very low in calcium and fortified with tricalcium phosphate or calcium carbonate to maintain the daily requirement. Oat milk, rice milk, and almond milk contain ≥ 50 mg/kg of calcium naturally, while cow milk is rich in calcium, which is 1050–1150 mg/kg.

Spelt beverages stand out for their abundant niacin content, while almond beverages supplemented with vitamins B12 and D2 excel in these specific nutrients. Soy beverages provide higher quantities of folic acid and essential minerals, surpassing milk and other plant-based alternatives, excluding salt, iodine, and chloride [6,20,21,22,23]. Hemp beverages are notable for their substantial contributions of manganese, copper, and selenium while retaining a salt content comparable to almond beverages [6]. Oat-based beverages lead in dietary fiber content, making them an excellent choice for those seeking a high-fiber option. For individuals concerned about iron intake, almond-based beverages contain the highest iron levels, whereas these nutritional variations allow consumers to make informed choices based on their dietary needs and preferences, highlighting the diversity in plant-based beverage options [21,23]. Soy milk contains an average of 5.93 mg/kg, 200 mg/kg, and 3.4 mg/kg of iron, magnesium, and zinc, respectively. In the case of vitamin B12 and vitamin D, most of the plant-based beverages are found to have limited in the quantity of these micronutrients, ranging from only 0–0.2 µg/100 mL. With the highest quantities of vitamins B1, B2, B6, folic acid, and vitamins E and K1 (phylloquinone), soy beverages stand out as the nutritional powerhouse among plant-based beverages, as reviewed here. Additionally, the soy and cashew beverages included sizable amounts of vitamin K1 [6,24]. However, the high content of microelements and essential nutrients in plant-based beverages may not have good bioavailability, as compared to other sources. This analysis highlights the nutritional diversity among different milk alternatives, providing options for varying dietary needs and preferences.

The energy (kcal/100 mL) of most plant-based beverages is less than cow’s milk, except for a few types of beverages under the category of nuts, such as almond milk, coconut milk, cashew nut, and rice-based beverages. This might be due to the presence of more fat content in nut-based beverages and a greater amount of carbohydrates in rice-based beverages. One-third (27.87%) of the total beverages observed were found to be higher in carbohydrate content compared to cow’s milk. Regarding the difference in composition, the carbohydrate content in cow’s milk is mostly lactose, which contributes to vitamin D and facilitates the absorption of calcium, phosphorus, and magnesium in the intestine, while plant-based beverages are lactose-free [4]. Out of all plant-based beverages, only 16.39% of the samples contain higher protein content than cow’s milk; that is, 0.10–0.97 g/100 mL in rice-based beverages and highest in soy-based beverages (0.99–4.49 g/100 mL). Regarding the fat or lipid content of plant-based beverages, only 13.11% of the plant-based beverages were reported to have a higher content of lipid or fat content compared to cow’s milk. Among the commonly studied plant-based beverages, the fat content varies; for instance, rice-based beverages have the lowest fat content, ranging from 0.00 to 1.95 g/100 mL, while coconut-based beverages have the highest fat content, ranging from 0.90 to 19.00 g/100 mL. Out of the plant-based beverages with available dietary fiber data, the majority (81.82%, n = 18) exhibit a higher dietary fiber content compared to the reference value of cow’s milk (0.00 g/100 mL) [4].

Iron is another essential micronutrient, required for the formation of red blood cells, wound healing, providing energy to the body, maintaining immunity, and carrying oxygen throughout muscles. Cow milk contains little or no iron, and the other plant-based beverages are very low in iron and need to be fortified. Cow milk contains 100 mg/kg of magnesium and 3.42 mg/kg of zinc, which are essential for healthy muscles and bones, and protect against cardiac attack, diabetes, and osteoporosis. Soy milk, almonds, and cashews are better sources of minerals, such as iron, magnesium, and zinc, compared to rice and coconut milk [6].

**Table 2:** Reported mean values and range (minimum–maximum) of micronutrients in selected plant-based beverages.

Type of Beverages *	Calcium (mg/kg)	Iron (mg/kg)	Magnesium (mg/kg)	Zinc (mg/kg)	Vitamin B_12_ (µg/100 g)	Vitamin D (µg/100 g)	References
Cow milk (n = 2)	1121 (1090–1150)	nd	100 (100)	3.42 (3.37–3.48)	0.2	nd	[6,20]
Soy milk (n = 7)	842 (80–1670)	5.93 (3.29–9.86)	200 (130–270)	3.4 (2.4–4.43)	0.1 (0.0–0.3)	0.4 (0.0–1.0)	[6,20,22,23]
Oat milk (n = 4)	499 (20–1330)	0.83 (0.0–1.94)	42 (20–70)	0.28 (0.0–0.53)	0.1 (0.0–0.3)	0.3 (0.0–1.1)	[6,20]
Rice milk (n = 5)	544 (50–1040)	1.42 (0.0–2.42)	68 (30–100)	0.53 (0.4–0.73)	nd	nd	[6,20,23]
Almond milk (n = 4)	656 (50–1250)	1.21 (0.72–2.22)	95 (60–170)	1.33 (0.62–2.74)	0.2 (0.0–0.6)	0.4 (0.0–1.2)	[6,20,21,23]
Cashew milk (n = 2)	64 (60–70)	2.95 (1.86–4.04)	158 (110–210)	3.04 (1.8–4.28)	nd	nd	[6]
Coconut milk (n = 3)	471 (30–1330)	0.62 (0.31–0.86)	59 (30–90)	0.36 (0.24–4.23)	0.03 (0.0–0.1)	0.2 (0.0–0.5)	[6,20,23]
Hemp milk (n = 1)	45	2.08	76	1.49	nd	0.2	[6]

* n is the number of studies reporting the composition data.

## 3. Fatty Acid Profile of Plant-Based Beverages

Several studies have investigated the fatty acid profiles of various plant-based beverages, including soy, oat, coconut, almond, and rice milk. Cow’s milk primarily contains saturated fatty acids in its lipid composition, whereas plant-based beverages are devoid of cholesterol and typically have a higher proportion of unsaturated fatty acids, except in coconut-based beverages [15].

Almond milk is rich in monounsaturated fats, primarily oleic acid. It also contains small amounts of polyunsaturated and saturated fats [25]. Soy milk is a good source of polyunsaturated fats, particularly linoleic acid (an omega-6 fatty acid), and contains some monounsaturated and saturated fats. Soy milk is reported to contain approximately 15% saturated and 80% unsaturated fatty acids [26]. Coconut milk contains minimal amounts of unsaturated fats but is high in saturated fats, mainly lauric acid [27]. Oat milk contains mainly unsaturated fatty acids, such as oleic and linoleic acids [28]. The ratio of unsaturated to saturated fatty acids is relatively high. The range of different fatty acid contents in different genotypes of oats was studied and it was found that, among the three major fatty acids, significant differences were mostly found in oleic acid rather than in palmitic and linoleic acids. Lidia (Swedish genotype), Ivore, and Dakar (French genotype) oats contained the highest quantities of palmitic, oleic, and linoleic acids, respectively [28]. Rice milk appears to be low in fat compared to other plant-based milk products. It primarily contains unsaturated fatty acids, with linoleic acid being a prominent component [29]. Another study highlights the composition and potential benefits of plant-based milk derived from kidney beans, specifically two local varieties grown in Turkey. The majority of the fatty acids in the kidney bean milk samples were α-linolenic acid (25.66–27.78%) and palmitic acid (18.95–23.08%). These studies provide insights into the fatty acid profiles of various plant-based milk alternatives, which can be valuable for individuals seeking specific nutritional components in their diets. The fatty acid composition can vary based on factors such as processing methods, varieties of the plant, and additives used in the beverages [2].

Table 3 outlines the omega-3 fatty acid content of selected plant-based beverages. Hemp seed beverage appears to be the best source of omega-3 fatty acids among the listed beverages, with the highest content of 16.50 g/100 g total fatty acids. Soy milk also provides a substantial amount of omega-3 fatty acids at 7.40 g/100 g total fatty acids. Oat and cow’s milk contain a small amount of omega-3 fatty acids compared to hemp and soy drinks [15,20,30,31,32]. Almond, hazelnut, rice, and coconut beverages have negligible or undetectable amounts of omega-3 fatty acids [16].

## 4. Fortification of Plant-Based Beverages to Overcome Nutrient Deficiencies

Plant-based beverages are often misunderstood as healthy alternatives to dairy but present some challenges in meeting certain nutrient requirements. Different studies have recorded potential nutrient deficiencies in individuals consuming plant-based beverages. These include deficiency of protein, vitamin D, vitamin B12, omega-3 fatty acids, iron, calcium, iodine, and zinc [33,34,35].

The deficiencies of different plant-based beverages have been studied by different researchers. Almond-based beverages have lower protein concentration than some other plant-based beverages, but it is frequently consumed for their creamy texture and nutty flavor rather than the protein content. Rice-based beverages, especially those produced from broken polished rice, often have lower levels of iron and magnesium due to the losses during processing. Furthermore, among plant-based milk substitutes, choices made from rice often have the lowest salt level. Coconut-based beverages, on the other hand, have very low dietary fiber value and calcium levels, which may not make them the best option for anyone interested in having these nutrients. The beverages made from sunflower seeds and hemp seeds stand out as having a low carbohydrate level, which may appeal to people looking for a lower-carb substitute. Because these nutritional characteristics may differ significantly between brands and formulas, it is crucial to read labels to make educated dietary decisions [4]. Plant-based beverages are frequently fortified to increase their dietary content and include critical vitamins and minerals that may be missing in the raw materials. When compared with cow’s milk, plant-based beverages such as almond, soy, or rice milk are frequently poorer in nutrients such as calcium, vitamin D, vitamin B12, and sometimes protein. Companies may fortify these beverages to render them more nutritionally comparable to dairy milk and guarantee that customers have a source of critical nutrients, which is especially crucial for vegan or dairy-free individuals. For customers who choose plant-based alternatives, fortification assists in tackling any nutrient deficits and fosters improved overall nutritional health [36].

Though plant-based beverages are good sources of different nutrients, they are found to be deficient in certain essential nutrients like omega-3 fatty acids. As plant-based beverages are popularly available in the market and consumers also prefer to have these products over beverages from animal sources, these beverages may be upgraded with certain essential fatty acids, which can have a positive health benefit among populations in terms of addressing certain non-communicable diseases like hypertension, cardiovascular diseases (CVDs), and cancer.

A well-planned vegan or vegetarian diet is considered to provide all the essential nutrients for good health. However, there are concerns about the risk of inadequate nutrient intakes, such as calcium, vitamin D, vitamin B12, and n3 PUFAs (particularly EPA and DHA) in unplanned and/or unfortified vegan or vegetarian diets [37]. Because the human body cannot naturally produce these valuable PUFAs, it must rely on omega-3 fortified food products to reap some of the many health benefits, including improved cognitive capacity, memory, and brain function, increased bone density, decreased risk of osteoporosis, reduced joint pain and stiffness, lowered inflammation, decreased risk of cardiovascular disease and stroke, and improved heart health [38]. The plant-based beverages have distinctive tastes and aroma profiles, which must be protected from oxidative changes or the development of undesirable odors in fortified PUFAs [39]. It becomes a subject of public health concern to ensure that the new plant-based beverage products do not have a lower nutritional content than milk but have enough nutritional value. Regulatory authorities, standardization groups, the food sector in the US and the EU, and the worldwide population might benefit from the creation of a set of recommended nutritional standards for the developing plant-based beverages category. Such guidelines would highlight optimal manufacturing practices and/or any future plant-based beverages’ nutritional content claims when applied to both new and current plant-based beverages. Only voluntary nutrient profiles and front-of-pack labeling have been connected to nutritional needs for the plant-based product category thus far. Plant-based beverages are one of the requirements for beverages listed by the European Nutri-Score, Australia–New Zealand Health Star Rating, and Choices International to receive a high score [36].

Plant-based beverages have been fortified by manufacturers in the US and across the world with nutrients that are closely related to those in milk, but occasionally in variable proportions. For instance, soy milk in the US is consistently fortified with calcium, vitamin A, and vitamin D in amounts equivalent to milk, and has a reasonably high protein content (3/100 g) and high PDCAAS value (>0.90). The US Department of Agriculture consequently places fortified soy milk in the dairy group, as opposed to other plant-based beverages. In the US, dairy milk is vitamin D-fortified; in other nations, this may not be the case [36].

In the UK, plant-based products are becoming more popular, particularly among young women, which may have an impact on iodine consumption because they are inherently low in iodine; this is worrisome because iodine is necessary for fetal brain development. Plant-based option sales in the UK were 73% higher in 2020 than in 2018. Plant-based dairy alternatives have seen the most significant growth in the UK market in recent years, with sales of plant-based milk and cheese alternatives increasing by 107% and 165%, respectively, in 2020 compared with 2018 [40].

Among the demographic, young women are notably propelling this shift, with 26% of them opting for plant-based beverage replacements. Moreover, a substantial portion of the 16–24 age group, up to one-third, is showing a preference for these plant-based alternatives. However, this growing inclination poses a challenge concerning iodine intake. The raw materials used for producing alternatives to fish and dairy inherently lack iodine and do not provide this essential nutrient unless fortified. A prior study on milk alternatives in the UK in 2015 revealed that a mere 6% of the milk replacements available in the market were iodine-fortified [40].

## 5. Fortification of Omega-3 Fatty Acids in Plant-Based Beverages and Health Benefits

Omega fatty acids are a form of fat that may be obtained from food or supplementation. Based on their chemical structure, they are divided into three primary categories: omega-3, omega-6, and omega-9. The omega-3 polyunsaturated fatty acids (PUFA), including alpha-linolenic acid (ALA; 18:3–3), docosapentaenoic acid (DPA; 22:5–3), DHA (22:6–3), and, to a lesser extent, EPA (20:5–3), have been found to have positive effects on health. Omega-3 fatty acids are abundant in marine and plant sources (for example, linseed oil and rapeseed oil) and are most commonly extracted from fish, marine animals, algae, or flax. Fish oils from anchovies or cod liver are the traditional sources of omega-3 fatty acids, but more recently, omega-3 fatty acids have also been derived from krill, squid (or calamari), algae, and other sources [41].

Omega-3 fatty acids are the essential fatty acids that are critical for maintaining overall health and well-being. The different evidence-based studies reported on the health benefits of these fatty acids for overall health status. It has been reported that omega-3 fatty acids improve vascular tone, heart rate, blood cholesterol levels, inflammatory reactions, blood pressure, and less arterial hardening. The anti-inflammatory effects of omega-3 fatty acids are primarily responsible for their health benefits. Atherosclerosis, chronic hepatitis, inflammatory bowel conditions, psoriasis, and rheumatoid arthritis can all be treated with omega-3 fatty acids [42].

Oat-based beverages have gained significant attention in the market, portraying potential health benefits for cancer patients [21]. Oats are unique among cereals, offering easily digestible protein, good fats (unsaturated fatty acids), essential minerals, vitamins, antioxidants, and dietary fiber called beta-glucan. Beta-glucan helps control blood sugar, lowers cholesterol, and reduces the risk of heart disease. These qualities make oat drinks a nutritious choice, particularly beneficial for people going through cancer treatment [20,43].

The increasing use of foods and supplements enriched with these important fatty acids is driven by consumer awareness of the health advantages of omega-3 fatty acids. Microalgae are regarded as a hopeful and environmentally friendly reservoir of omega-3 polyunsaturated fatty acids. Particularly, the microalgae belonging to the *Schizochytrium* genus are extensively employed components in feeds for aquaculture [44]. According to reports, 38% of customers specifically search for omega-3 fatty acids while making food and drink purchases. Numerous studies have demonstrated the benefits of omega-3 fatty acids in preventing heart disease, lowering blood pressure, lowering the risk of stroke and certain cancers, managing diabetes and inflammatory diseases, assisting in weight loss, and improving calcium absorption, skin healing, and mental clarity [42,43,45].

## 6. The Challenges and Recommendations for the Fortification of Omega-3 Fatty Acids in Plant-Based Beverages

Fortifying plant-based beverages with omega fatty acids presents various challenges that need to be addressed to ensure the successful incorporation of these essential nutrients. Omega fatty acids, particularly omega-3 and omega-6, are crucial for human health and are commonly found in fish and other animal products. However, fortifying plant-based beverages with these fatty acids can be challenging due to several reasons like compatibility and stability, taste and sensory acceptance, bioavailability, cost and sourcing, regularity compliance, formulation and processing, etc. [46].

Plant-based beverages that have distinctive tastes of their own are a crucial consideration in this situation. Other components, like fish oil, become more challenging to incorporate. The biggest issue with using omega-3 fatty acids in food is oxidation because of their inherently unpleasant fishy flavor. The industry has created a wide range of novel antioxidants and encapsulating techniques to avoid oxidation and off odors [39].

Utilizing fish oil, either in liquid form (containing 30% EPA and DHA) or as a microencapsulated powder (with 10% content), can enhance the nutritional value of functional food beverages. However, Kolanowski and Berger [47] cautioned that incorporating fish oil into low-pH water-based products like fruit drinks can accelerate hydrolysis and oxidation, consequently reducing the shelf life of the final product. Fortifying liquid products like milk or vegetable juices with fish oil, even at a significant level of up to 0.1%, provides 0.03% EPA and DHA and adversely affects palatability. This indicates that enriching mild-flavored and mildly sweet liquids with fish oil may not be ideal due to the resultant loss of taste.

Omega-3 fatty acid fortification in plant-based beverages is crucial because it enhances their nutritional profile. These fatty acids, particularly EPA and DHA, offer numerous health benefits, such as supporting heart and brain health. Fortifying plant-based drinks with omega-3 provides these benefits to individuals who follow a vegetarian and vegan diet. It also provides dietary variety and caters to those with sensitivities or dietary restrictions who cannot consume fish-derived sources of omega-3. Moreover, as plant-based diets gain popularity, there is a growing demand for fortified products, making this a strategic move for businesses to tap into a burgeoning market of health-conscious consumers. Omega-3 fortification in plant-based beverages contributes to improved nutrition, health advantages, dietary inclusivity, market competitiveness, and consumer satisfaction.

Hence, just as with any dietary supplement, it is essential to customize one’s daily omega-3 intake to meet their unique health requirements. At the same time, regulatory authorities in different countries need to approve these supplements to ensure their safety [48]. Moreover, individuals with seafood allergies should be especially cautious, and those who exhibit hypersensitivity to specific formulations should avoid their consumption. While omega-3 supplements generally do not appear to interact negatively with statins, excessive consumption can potentially lead to adverse effects on the immune system and blood clotting. Notably, a recent study has suggested that EPA/DHA supplements may contribute to delayed viral clearance and increased oxidative stress [49]. Therefore, a better selection of omega-3 fatty acids for fortification should be made. Other recommendations, such as optimizing the growth conditions of omega fatty acids and conducting sensory evaluations of fortified beverages, need to be followed [50].

## 7. Conclusions

The rising popularity of plant-based beverages, driven by health, ethical, and environmental concerns, has given rise to a diverse range of products. These beverages offer unique nutritional profiles but may also present certain nutrient deficiencies, including omega-3 fatty acids. To address these deficiencies and provide consumers with the associated health benefits, the fortification of plant-based beverages with omega-3 fatty acids is a promising strategy. This practice not only caters to the growing market of health-conscious individuals but also ensures that these beverages remain nutritionally robust. However, the process of fortification comes with its challenges, including issues related to taste, stability, and sourcing sustainable ingredients. It is crucial to consider individual health needs and ensure regulatory oversight to guarantee the safety and efficacy of these fortified products. Fortifying plant-based beverages with omega-3 fatty acids is a proactive step in bringing a more comprehensive and nutritionally balanced range of product choices to consumers, aligning with their evolving dietary preferences and health-conscious lifestyles.

## Figures and Tables

**Table 1:** Calories and nutrient content (range) of various plant-based beverages under development or commercially available.

Type of Beverages *	Energy (kcal/100 mL)	Carbohydrate (g/100 mL)	Protein (g/100 mL)	Fat (g/100 mL)	Dietary Fiber (g/100 mL)	References
Cow’s milk (n = 1)	64.00	4.65	3.28	3.66	-	[15]
Soy milk (n = 34)	31.00–69.00	0.10–12.41	0.99–4.49	0.51–3.17	0.50–1.33	[15,16,17,18]
Oat milk (n = 7)	36.00–53.00	1.00–8.90	0.40–1.86	0.37–2.69	1.00–4.40	[4,16,17,18]
Rice milk (n = 11)	18.00–66.00	2.57–12.84	0.10–0.97	0.00–1.95	0.00–1.09	[18,19]
Almond milk (n = 12)	12.00–68.00	0.10–4.50	0.31–4.36	1.10–5.51	0.20–2.16	[4,16,17,18,19]
Cashew milk (n = 8)	23.00–79.00	2.60–5.73	0.42–2.20	1.04–5.29	0.20–1.18	[4,15,19]
Coconut milk (n = 8)	20.00–183.00	0.51–9.41	0.10–2.00	0.90–19.00	-	[4,16,17,18,19]
Hemp milk (n = 3)	19.00–40.00	0.10–2.50	0.10–1.00	1.25–2.90	-	[16,17,18]

* n is the number of samples included for the data.

**Table 3:** Omega-3 fatty acid content of different plant-based beverages.

Type of Beverages	Total Omega-3 Fatty Acids (g/240 mL)	Total Omega-3 Fatty Acids (g/100 g)	References
Cow’s milk	0.2	0.43 g/100 g	[16,30,32]
Soy milk	0.2	7.40 g/100 g	[16,30,31]
Oat milk	0.1	1.71 g/100 g	[16,30]
Rice milk	<0.01	Not detected	[16]
Almond milk	0.05	0.14 g/100 g	[16,30]
Cashew milk	0.02	Negligible	[16]
Coconut milk	0.01	Not detected	[16]
Hemp milk	1.0	16.50 g/100 g	[30]

## Data Availability

No new data were created or analyzed in this study.
